# The Role of the Tumor Microenvironment in Pancreatic Ductal Adenocarcinoma: Recent Advancements and Emerging Therapeutic Strategies

**DOI:** 10.3390/cancers17101599

**Published:** 2025-05-08

**Authors:** Franciszek Glapiński, Weronika Zając, Marta Fudalej, Andrzej Deptała, Aleksandra Czerw, Katarzyna Sygit, Remigiusz Kozłowski, Anna Badowska-Kozakiewicz

**Affiliations:** 1 Students’ Scientific Organization of Cancer Cell Biology, Department of Oncological Propaedeutics, Medical University of Warsaw, 01-445 Warsaw, Poland; 2 Department of Oncological Propaedeutics, Medical University of Warsaw, 01-445 Warsaw, Poland; marta.fudalej@wum.edu.pl (M.F.); andrzej.deptala@wum.edu.pl (A.D.); 3 Department of Oncology, National Medical Institute of the Ministry of the Interior and Administration, 02-507 Warsaw, Poland; 4 Department of Health Economics and Medical Law, Medical University of Warsaw, 02-091 Warsaw, Poland; aleksandra.czerw@wum.edu.pl; 5 Department of Economic and System Analyses, National Institute of Public Health NIH—National Research Institute, 00-791 Warsaw, Poland; 6 Faculty of Health Sciences, Calisia University, 62-800 Kalisz, Poland; 7 Department of Management and Logistics in Healthcare, Medical University of Lodz, 90-131 Lodz, Poland

**Keywords:** pancreatic ductal adenocarcinoma, microenvironment, stellate cells, cancer-associated fibroblasts, tumor-associated macrophages, neuroinvasion, stroma, microbiome

## Abstract

Pancreatic cancer (PC), with pancreatic ductal adenocarcinoma (PDAC) comprising about 90% of all cases, is one of the most aggressive and lethal solid tumors. PDAC remains one of the most significant challenges of oncology to this day due to its inadequate response to conventional treatment, gradual rise in incidence since 2004, and poor five-year survival rates. As cancer cells are the primary adversary in this uneven fight, they remain the primary research target. Nevertheless, increasing attention is being paid to the tumor microenvironment (TME). The most crucial TME constellation components are immune cells, especially macrophages, stellate cells and lymphocytes, fibroblasts, bacterial and fungal microflora, and neuronal cells. Depending on the particular phenotype of these cells, the composition of the microenvironment, and the cell ratio, patients can experience different disease outcomes and varying vulnerability to treatment approaches. This study aims to present the current knowledge and review the most up-to-date scientific findings regarding the microenvironment of PC. It contains detailed information on the structure and cellular composition of the stroma, including its impact on disease development, metastasis, and response to treatment, as well as the therapeutic opportunities that arise from targeting this tissue.

## Simple Summary

Pancreatic cancer (PC) remains one of the most significant challenges of oncology to this day due to its inadequate response to conventional treatment, gradual rise in incidence, and poor five-year survival rates. Increasing attention is being paid to the tumor microenvironment (TME), which comprises a diverse array of cell types. The TME potentially brings a new turn in PC survival. This study aims to present the current knowledge and review the most up-to-date scientific findings regarding the microenvironment of PC. It contains detailed information on the structure and cellular composition of the stroma, including its impact on disease development, metastasis, and response to treatment, as well as the therapeutic opportunities that arise from targeting this tissue.

## 1. Introduction

Pancreatic cancer (PC), with pancreatic ductal adenocarcinoma (PDAC) comprising about 90% of all cases, is one of the most aggressive and lethal solid tumors [1]. PDAC derives from ductal-like cells, which may originate from mutated ductal epithelial cells or acinar cells that undergo metaplasia to adopt a ductal phenotype. These processes are often triggered by stressors such as alcohol and nicotine consumption, chronic inflammation, diabetes, and poor nutrition [2]. Driver mutations include activating mutations of protooncogene KRAS and the inactivation of tumor suppressor genes such as SMAD4, CDKN2A, and TP53 [3]. Pancreatic cancer typically develops from well-recognized precursor lesions, such as pancreatic intraepithelial neoplasia (PanIN), intraductal papillary mucinous neoplasms (IPMN), and mucinous cystic neoplasms (MCN). Pancreatic cancer remains one of the greatest challenges in oncology, driven by its increasing incidence, lack of reliable biomarkers, and late detection, which together contribute to poor treatment responses and low five-year survival rates [4]. Moreover, PDAC features a dense stromal microenvironment characterized by notable cellular and spatial variation, which plays a crucial role in shaping disease behavior and resistance to treatment [5]. According to the Surveillance, Epidemiology, and End Results Programme (SEER) Database, more than 67,000 new cases are expected in the United States alone. It will constitute 8.4% of all cancer deaths (almost 50,000 deaths per year), and the 5-year survival rate will be equal to only 13.5% [6]. According to the Polish National Cancer Registry, the incidence of PDAC in Poland increased by 18%, and mortality rose by 45% between 1996 and 2018. It is predicted that, by 2030, pancreatic cancer may become the second leading cause of cancer-related deaths in Poland [7]. Even though surgical resection with negative margins and adjuvant chemotherapy that target cancer cells is proven to be quite successful, in most cases, either the tumor is diagnosed at an unresectable stage, or the chance of recurrence is high, affecting the 5-year survival rate [3]. The first-line treatment chemotherapy scheme used in PDAC is a combination of fluorouracil, leucovorin, irinotecan, and oxaliplatin, known as FOLFIRNOX. The other scheme is gemcitabine associated with nab-paclitaxel. [8]. The roles of radiotherapy and immunotherapy are still being discussed, as they have not been validated by a substantial number of fully randomized trials [9]. There was a study that attempted to divide PDAC patients into subgroups based on CT images for the adjustment of treatment decisions for each patient, but the results are inconclusive, with more research required to reach a consensus. PC, even when resectable (R-PDAC) or borderline-resectable (BR-PDAC), should be presumed to be a systemic disease, and all patients should receive multimodal treatment [10]. Nevertheless, less than half of the patients respond to therapy, and they could suffer from the side effects of chemotherapeutics [11]. Genetics might explain the difference between respondents and non-respondents, but more evidence is required to use it in clinical settings [12]. Given its dramatic influence on cancer development and progression, it has prompted the exploration of novel strategies to target the tumor microenvironment rather than the parenchyma [13].

The aim of this work is to present the current knowledge and review the most up-to-date scientific findings regarding the microenvironment of PCs. It contains detailed information on the structure and cellular composition of the stroma, including its impact on disease development, metastasis, and response to treatment, as well as the therapeutic opportunities that arise from targeting this tissue.

## 2. The Structure of the Microenvironment

PC’s tumor microenvironment (TME), also known as its stroma, is responsible for maintaining and stabilizing the parenchyma, which is composed of cancer cells [14]. It is characterized by desmoplasia, a process in which dense fibrous connective tissue replaces the normal parenchyma of the organ. It is the primary pathophysiological reason for the pancreas’s malfunction during the progression of PDAC [15]. The stroma itself can represent up to 90% of a tumor mass. It is formed by cellular components such as stellate cells (PSCs), which can constitute more than 50% of the mass of the stroma or cancer-associated fibroblasts, as well as non-cellular components, which is mainly composed of the extracellular matrix (ECM), made up of collagens, glycosaminoglycans, and proteoglycans [13,16]. Additionally, immunological components could be present, mainly in the form of regulatory T lymphocytes (Treg lymphocytes), tumor-associated macrophages (TAMs), and myeloid-derived suppressor cells (MDSCs), together with cytokines, growth factors, and ECM-metabolizing enzymes secreted by those cells; the cells mentioned above present immunosuppressive functions [17,18]. The components of the TME maintain a connection with cancer cells indirectly, influencing tumor progression, metastasis development, and treatment resistance [19].

### 2.1. Pancreatic Stellate Cells (PSCs)

PSCs are star-shaped cells presented physiologically in the exocrine part of the pancreas in periacinar, perivascular, and periductal spaces in a quiescent state. Their proliferation rate is low, and they produce minute amounts of ECM. They are characterized by nestin expression and are involved in storing vitamin-A-rich lipid droplets, antigen phagocytosis, and the induction of amylase secretion [20]. They become activated during inflammation or the development of pancreatic tumors due to exposure to cytokines and growth factors produced by evolving cancer cells, inflammatory cells, acinar cells, platelets, and later by newly activated stellate cells, which means that their active state is maintained by paracrine and autocrine mechanisms [17]. Previously mentioned cytokines and chemokines include platelet-derived growth factor (PDGF), fibroblast growth factor (FGF), transforming growth factor β (TGF–β), tumor necrosis factor-alpha (TNF-α), connective tissue growth factor (CTGF) and interleukin 1 (IL-1), IL-6, IL-8, and IL-10 [16,21,22]. The cells secreting individual factors responsible for activating stellate cells are presented in Table 1.

Other factors contributing to the activation of PSCs include hyperglycemia, hypoxia, hypoperfusion, and oxidative stress, which often result from chronic inflammation [16,23]. Additionally, ethanol, its metabolites, and cigarette smoking are also considered risk factors for PDAC development [16,23]. After activation, PSCs begin to express a phenotype closely resembling that of myofibroblasts, as supported by the expression of α-smooth muscle actin (αSMA), desmin, and the loss of lipid droplets [24]. PSCs’ primary role is to deposit ECM components such as collagens, fibronectin, laminin, or hyaluronic acid. Their other functions include promoting cancer cell proliferation, migration, and apoptosis inhibition [25]. Those processes are promoted by specific cytokines and growth factors such as iIL-1 or IL-6, and IL-8, insulin-like growth factor 1 (IGF1), vascular endothelial growth factor (VEGF), PDGF, FGF, CTGF, and C-X-C motif chemokine 12 (CXCL12) [11]. Tumor necrosis factor α (TNFα), also secreted by the MDSCs and M1 macrophages described later, is theorized to play a role in the cachectic effects associated with tumor progression [26]. Those molecules lead to the intensification of angiogenesis processes and the proliferation and migration of parenchymal cells, ultimately resulting in metastasis formation. It has also been postulated that PSCs are responsible for chemotherapy and radiotherapy resistance [27,28]. The importance of investigating these mechanisms is high, as patients with PDAC who develop irreversible cachexia have a life expectancy below 3 months [29].

### 2.2. Cancer-Associated Fibroblasts (CAFs)

CAFs are a heterogeneous group of spindle-shaped cells of mesenchymal origin (classified due to mesenchymal marker vimentin) in the connective tissue of nearly all body organs, including the pancreas [30]. They are another class of cells responsible for ECM deposition and the formation of PDAC’s stroma [31]. There are various theories regarding the origin of CAFs in the pancreatic stroma. They may be the population of cells in the stroma that arise from local fibroblasts, mesenchymal stem cells, vascular smooth muscle cells, adipocytes, or even hematopoietic stem cells. Still, they may also be derived from bone marrow fibrocytes that differentiate into fibroblasts and migrate to the organ thanks to the cytokines and chemokines released by PSCs and cancer cells [15,30,32,33]. Cytokines responsible for the attraction, activation, and proliferation of CAFs include TGF-β, FGF, CTGF, and PDGF [32]. Another theory suggests that cells differentiate from active forms of PSCs, and if activated forms of these cells are present in cancer tissue, they should be referred to as CAFs [34]. This is because they not only express fibroblastic markers such as fibroblast activation protein (FAP) and fibroblast-specific protein 1 (FSP1), but PSC’s markers as well, like desmin and, most abundantly, αSMA [32,34]. The third theory proposed by Iwano et al. (2002) suggests that CAFs originate from endothelial cells through an endothelial–to–mesenchymal transition (EMT). This mechanism may explain the presence of these cells near the tumor, as the abundance of growth factors such as TGF-β, EGF, and FGF-2 drives this change, while only a few endothelial cells are present [35]. Due to various subtypes of CAFs, the theories mentioned above may not be exclusive. Öhlund et al. (2017) described the presence of two distinctive subtypes. The first subtype is a myofibroblastic subtype (myCAF), classified by a high concentration of α-SMA. In contrast, the second subtype, characterized by low αSMA expression, is referred to as the inflammatory subtype (iCAF) and exhibits a distinct pattern of IL–6 expression which is entirely absent in myCAFs [34]. MyCAFs are cells located in the proximity of the tumor, similar to PSCs, with the same functions of ECM protein synthesis, deposition of connective tissue, and promotion of neovascularization mainly through TGF-β, stromal-derived factor-1 (SDF-1), PDGF, and VEGF. In contrast, iCASs are identified at distant locations from the tumor and are responsible for secreting inflammatory cytokines, the most abundant being IL-6 [30]. Other cytokines and chemokines include IL-8, CXCL12, and C–C motif chemokine 2 (CCL2) [34,36]. All of them can induce tumor growth, cell invasion, and proliferation, with IL–6 also being responsible for angiogenesis [30]. The third population, identified by Elyada et al. (2019), is referred to as antigen-presenting (apCAFs) due to the presence of MHC II and CD74. ApCAFs present antigens to T cells, but also have other functions and roles in tumor development [37].

#### How Cancer-Associated Fibroblasts Impact Tumor Behavior

The strong desmoplastic reaction was formerly appreciated as a defensive mechanism for restraining tumor growth, similar to the one in chronic pancreatitis. Several years ago, fibroblasts were shown to suppress the growth of several cell lines, a phenomenon known as neighbor suppression, suggesting their protective role. Different types of fibroblasts develop a spectrum of suppressive activity. For example, skin fibroblasts appear to have stronger inhibitory effects than those obtained from prostate, nasal polyps, or inguinal hernias [38]. Nevertheless, the optics have shifted over the last 15 years, and fibroblasts’ impact now appears to be more ambiguous [39].

CAFs are significant contributors to PDAC progression due to their ability to promote fibrosis, which puts up a physical barrier preventing drug penetration. Inhibiting or depleting CAFs is a promising method undergoing numerous investigations [40].

One of these methods is the inhibition of the SLC7A11 protein. High stromal expression of SLC7A11 is correlated with poor prognosis, unlike the high tumor expression of SLC7A11, which does not predict patient survival [41]. SLC7A11 is a membrane transporter that transfers cysteine into cells in exchange for glutamate. It is essential in maintaining adequate fibroblast cell growth because it sits at the crux of many metabolic pathways, especially glutathione synthesis [42].

Due to KRAS mutation and metabolic ratio changes, both PDAC cells and CAFs are exposed to oxidative stress. Cancer cells require hypermetabolism to sustain growth, which results in the production of reactive oxygen species (ROS) in mitochondria, NADPH oxidases (NOXs), peroxisomes, and the endoplasmic reticulum (ER) [43,44,45]. Cancer cells are equipped with numerous antioxidant systems to counteract elevated levels of reactive oxygen species (ROS). Glutathione is the most abundant nonenzymatic ROS scavenger in living cells, and its production can be regulated by metabolic shifts [46].

Inhibition of SLC7A11 with sulfasalazine, an immunosuppressive medication commonly used to treat Crohn’s disease, significantly reduces PDAC growth. Interestingly, it does not work by inhibiting NF-κB, the main pathway interrupted when using sulfasalazine to treat inflammatory bowel disease [47], but rather by inhibiting the SLC7A11 transporter [41,48]. Additionally, direct inhibition of SLC7A11 using specific small interfering RNAs (siRNAs) has been shown to reduce PDAC tumor growth, metastasis incidence, CAF activation, and fibrosis in orthotopic mouse models of PDAC. It also decreases CAF proliferation and viability by inducing cysteine starvation [41].

Cancer cells could lose the Col1a2 gene and accelerate PDAC development by promoting CD4+ cells, which aggravate immunosuppression and deplete CD8+ cells. Typically, fibroblasts produce heterotrimeric α1/α2/α1 collagen I, but, in contrast, cancer cells often lose the Col1a2 gene and start producing homotrimeric α1/α1/α1 degenerated collagen. Although the copy number of the Col1a2 gene in PDAC cells is similar to human pancreatic nestin-expressing cells, there are distinct differences in methylation patterns, which are inversely proportional to protein expression. Deleting the Collagen 1 gene delays the development of acinar-to-ductal metaplasia and intraepithelial neoplasia, especially in the early stages of PC development [49].

**Figure 1:**
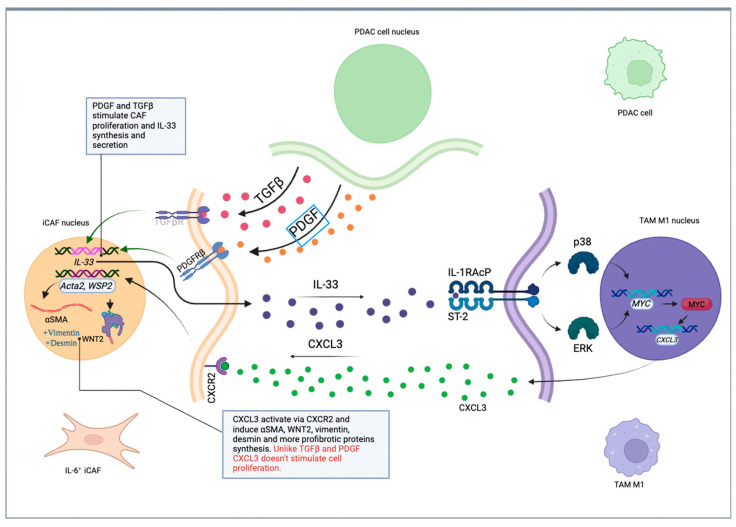
Scheme of mutual influence between PDAC cells, IL-6+ CAFs, and TAM M1 cells.

**Figure 2:**
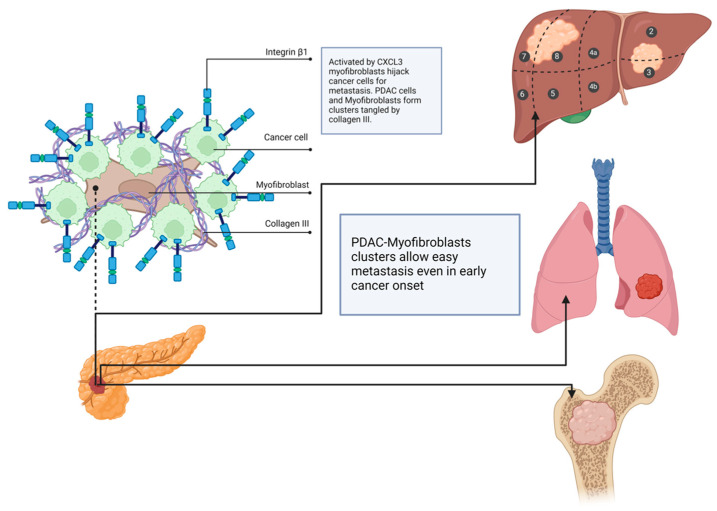
Scheme of PDAC, stroma cell clusters, and the most frequent metastasis locations (the liver is presented with subsegments according to the Couinaud system).

A new mechanism that facilitates PDAC metastasis has been recently discovered, highlighting the complex interactions between the stroma and cancer cells. PDAC cells, by secreting TGF-b, force endothelial cells and fibroblasts to produce IL-33. IL-33 works via the Il-33-ST2-p38/ERK-MYC-CXCL3 pathway and stimulates TAMs to produce CXCL3, which influences back fibroblasts by C–X–C–R motif chemokine 2 (CXCR2) on CAFs and turns them into myofibroblasts. In this manner, myofibroblasts can bind to collagen type 3 and form cell clusters, which can then migrate through the bloodstream and lymph vessels and metastasize (Figure 1 and Figure 2) [50].

The TME has a profound impact on the delivery of chemotherapeutic agents. The cellular component of PDAC is mainly made of alpha-smooth muscle actin (SMA+) expressing myofibroblasts [51]. SMA^+^ myofibroblasts associated with PDAC are thought to enhance collagen I production, thereby hindering drug penetration and T-cell infiltration [52]. The Sonic Hedgehog Homolog (SHH) pathway is a developmental morphogen contributing to stromal desmoplasia in various solid tumors. Absent in the adult pancreas, it reappears during inflammation and neoplasia. Interestingly, the SHH pathway has not been shown to contribute to normal differentiation of the pancreas. Ectopic administration of the SHH increases the number of mesenchymal cells [53,54]. Both the SHH ligand and its downstream signaling, including the transmembrane proteins Patched (Ptch) and Smoothened (SMO) [55], are induced in neoplastic lesions and increase during PDAC progression [53]. An elevated level of SHH induces a robust desmoplastic response in PDAC tumors by stimulating (αSMA+) fibroblast proliferation and chemotaxis, possibly through direct influence or by interacting with TGFβ signaling [56,57].

### 2.3. Extracellular Matrix

The most abundant protein in the body is collagen, represented mainly by types I and III. Still, other proteins that form the ECM are fibronectin and laminin. Other components include hyaluronic acid and a variety of proteoglycans, glycoproteins, and polysaccharides [15]. Olivares et al. (2017) hypothesize that collagen serves as the primary nutritional source for cancer cells. ECM proteins are visibly degraded, and the released amino acids, such as proline, may be utilized in the tricarboxylic acid cycle [58]. Enzymes involved in the degradation process are synthesized by PSCs and CAFs, which are matrix metalloproteinases 2, 3, 9, and 13 [59,60]. Their activation primarily relies on the concentration of TGF-β, which is secreted in significant quantities by CAFs [61]. ECM degradation (mainly collagenolytic) and remodeling are also responsible for tumor progression and invasion [60]. It has been suggested by Whatcott et al. (2017) that the predominance of specific ECM components correlates with the median survival of patients with PDAC. The median overall survival (OS) for patients with primary PDAC exhibiting strong stromal hyaluronic acid expression was 9.3 months, whereas it was 24.3 months for those with low hyaluronic acid staining [62]. Similarly, the same study revealed that low ECM type I collagen levels were associated with a median overall survival (OS) of 14.6 months, while high levels were associated with a median OS of 6.4 months [63].

### 2.4. Regulatory Lymphocytes (Treg Lymphocytes)

Treg lymphocytes are a subpopulation of T lymphocytes characteristic of the expression of CD4, CD25, and FOXP3 biomarkers. Their physiological role is to suppress the immune response. During tumor progression, they can actively suppress the anti-tumor immune response, indicating that they are essential for maintaining immunological self-tolerance [18,64]. It was reported that the percentages of CD4^+^CD25^+^FOXP3^+^ Tregs were significantly increased in PDAC tumor tissues compared to control pancreatic tissues. An increased abundance of tumor-infiltrating regulatory T cells is correlated with the progression and prognosis of pancreatic ductal adenocarcinoma]. According to Jang et al., a large proportion of these cells correlates with a poorer prognosis [18]. Their primary function is to release TGF-β and IL–10, which predominantly silence the immune response in the tumor’s proximity [65]. Several reports also state that Tregs hold a protective role, improving overall survival [66]. Further studies are needed to clarify the role of Tregs in PDAC and to determine how these findings might be translated into clinical management strategies for patients.

### 2.5. Myeloid-Derived Suppressor Cells (MDSCs)

MDSCs are defined as a heterogeneous group of immature myeloid cells originating in the bone marrow whose presence in other organs in the body almost always indicates chronic inflammation [67]. They are divided into two subtypes: the monocyte-resembling subtype (M–MDSC) and the polymorphonuclear-resembling subtype (PMN–MDSC) [68]. They are the most prominent group of immune cells in the TME of PDAC [65]. They are activated by cytokines present during inflammation, including granulocyte-macrophage colony-stimulating factor (GM-CSF) and IL–13.

Both populations of MDSCs are responsible for the evasion of anti-tumor immune response by contributing to forming an immune-privileged niche within the tumor microenvironment [69]. They activate Treg lymphocytes and suppress CD8+ T lymphocytes and NK cells [65,67]. This is performed by the EGFR-MAPK-dependent regulation of tumoral PD-L1 expression. They also secrete immunosuppressive molecules such as arginase-1, nitric oxide, and reactive oxygen species [70]. They are also one of the cells secreting cytokines responsible for the M2 differentiation of TAMs, which further amplify the pro-tumor effects of the TME [71]. Several studies in preclinical models have shown that depleting MDSCs or inhibiting their function enhances the anti-tumor response [72].

### 2.6. Tumor-Associated Macrophages (TAMs)

TAMs are cells derived from monocytes and are present in the pancreas under physiological conditions. They are responsible for the phagocytosis of various pathogens and act as antigen-presenting cells for T and B lymphocytes. There are two phenotypes of TAMs. M1, present in early tumor development, promotes inflammation and is capable of releasing TNFα, which is speculated to contribute to cachexia. The interaction of interferon-gamma receptor (IFN-γR), granulocyte-macrophage colony-stimulating factor receptor (GM-CSFR), and interleukin-12 receptor (IL-12R) on macrophages, with their respective ligands, induce M1-like polarization via activation of the Janus kinase (JAK)/signal transducer and activator of transcription (STAT) pathway [73]. It is generally considered to have anti-tumor properties, and their activity is suppressed by CAFs and tumor cells by secreting cytokines such as IL-8, IL-10, and TGF-β [74]. The microenvironment favors the M2 phenotype. The differentiation process into the M2 phenotype is promoted by IL-4, IL-10, IL-13, and TGF-β, which are produced by multiple components of TME [75]. These cytokines promote differentiation by activating STAT3/NF-κB pathway [76]. They exhibit pro-tumor effects, promote anti-inflammatory responses, and correlate with a poor prognosis of the disease [68,77]. It may be because they secrete proteins such as TGF-β that promote PDAC progression and metastasis [78].

### 2.7. Angiogenesis

The stroma in PDAC is considered to be hypovascular. Cancer cells and specific microenvironment components such as PSCs develop primarily in highly hypoxic conditions [79]. Moreover, the tumoral stroma has a distorted distribution, in contrast to the homogeneous evenly distributed spectrum of vessels in healthy organs, which is rich in microvessels and capillaries. This phenomenon is not only characterized by the increased prevalence of arteries and arterioles further from the parenchyma, but also by the phenomenon of vascular mimicry (VM) [80]. Cancer cells in some tumors can form vessel-like structures, further complicating the typical vascular architecture [81]. It becomes an independent tumor blood supply system that occurs without the involvement of angiogenesis or endothelial cells, and it is linked to poor survival in PDAC patients. EGF and VEGF, cytokines involved in the process of vascular mimicry, are thought to trigger enhanced Notch signaling. This activation subsequently leads to upregulating mesenchymal markers, such as TWIST1 and SNAI1, and facilitates the formation of tube-like structures characteristic of VM [82]. However, it must be highlighted that vasculature in the tumor’s proximity constantly changes during its progression. Changes in the ratio of proangiogenic to anti-angiogenic factors are hypothesized to trigger the angiogenic switch during pancreatic tumor formation [83]. Initially, the tumor progresses through its avascular phase, but as the tumor increases in size, it enters a vascular phase characterized by rapid angiogenesis, which is facilitated by cytokines secreted by PSCs and CAFs, such as VEGF and FGF, that promote SHH signaling and the proliferation of endothelial cells [84,85].

## 3. Stromal Cell Relations and the Influence of Connective Tissue on PDAC

Unlike any other malignancies, PDAC consists mainly of stromal cells (up to 90%) and only a minority of proper epithelial cancer cells. PDAC-associated stroma cells are stellate cells, macrophages, fibroblasts, and lymphocytes. Two of the most critical cells influencing PDAC survival and treatment outcomes are CAFs and TAMs, which alter the disease course [50]. The intense effort put into exploring stromal components fueled great optimism and resulted in the testing of numerous potential therapeutic targets [86]. Nevertheless, recent years provided data that the depletion of TME components may even exacerbate PDAC and contribute to the acceleration of metastasis and tumor growth by enhancing cancer cell proliferation [87,88,89].

### Epithelium–Stroma Relations: How Independent Is Stroma from Epithelium?

Using microdissection, primary reports studying concomitant tumor and stromal subtypes in PDAC revealed a partial dependence between epithelial and stromal molecular subtypes [51]. Up-to-the-minute convergent transcriptional tumor/stroma classification conducted in patient-derived xenografts (PDX) revealed two subtypes of PDAC with distinct outcomes. These subtypes uncovered specific alterations in DNA methylation, transcription, and signaling pathways involved in tumor-stromal cross-talk, e.g., hepatocyte growth factor (HGF), IGF, and SHH. Overall, the stroma, originating from surrounding host cells, is closely associated with the tumor phenotype, indicating that the composition and function of the TME may be influenced by tumor cells [90]. Mouse models of PDAC deficient in SHH demonstrated that Hedgehog inhibition leads to the absence of tumor stroma and the abundance of intratumoral blood vessels, yet increases tumor aggressiveness and metastasis [88].

Another essential factor in PDAC stroma development is retinoic acid (RA), which induces PSC deactivation and reduces extracellular matrix production by inhibiting the production of TβRII, PDGFRβ, β-catenin, and Wnt [91]. Further studies have demonstrated that RA-induced inhibition of EMT in tumor cells is mediated through the downregulation of IL-6 and CAFs [92]. CAFs–cancer-cell interactions in the PC organoid model could promote the EMT of cancer cells, which was reversed by the all-trans retinoic acid (ATRA)- mediated inhibition of CAFs [93].

High-mobility group A1 (HMGA1) chromatin regulators are epigenetic agents that could upregulate FGF19 expression, promote fibrotic tissue expansion, and induce desmoplasia [94].

Epithelium-stroma relations are mutual, as epithelium influences stroma, so stroma influences epithelium. CAFs could be responsible for acinar-to-ductal metaplasia (ADM) via the newly discovered laminin5/integrinα4/stat3 axis [95].

The stroma relies on cancer cells and other components of the PDAC constellation, including lymphocytes. Non-canonical CD8+ T-cell subpopulation producing IL-17A (Tc17) accelerates tumor growth via IL-17RA-dependent stroma modification, as IL-17A and TNF synergistically induce differentiation of inflammatory CAFs (iCAFs) by IL-17RA [96].

## 4. Neuronal PDAC Infiltration and Contribution to Metastasis

Pancreas innervation is both extrinsic and intrinsic. Massive innervation is necessary for regulating endocrine function and controlling the secretion of enzymes in response to nutrient intake. It also controls smooth muscle contractility, splanchnic microcirculation, and epithelial cell absorption [97]. Remarkably, pancreatic nerves undergo severe alterations during carcinogenesis. Intrapancreatic nerves grow in size and number. The proportion of autonomic and sensory fibers is switched, and perineural inflammatory cells, or PC cells, begin to infiltrate neurons [98]. The mechanism by which PDAC induces these changes in the neuronal environment remains unknown. Nerve hypertrophy is significantly distinct in areas with the strongest desmoplasia. It seems that PDAC produces factors necessary for nerve regeneration [99].

In general, the nerve–cancer relationship suggests a tumor-promoting relationship in PDAC. The first studies in this field revealed that neurons can increase the invasiveness and migration of cancer cells toward neuronal targets. Perineural invasion is a critical cause of local recurrence [100,101].

Although neuronal invasion occurs in most solid gastrointestinal tumors, the severity of this process is most pronounced in PDAC [102]. The formation and function of perineural invasion are regulated by molecular (e.g., involving neurotrophins, cytokines, chemokines, and neurotransmitters), metabolic (e.g., serine metabolism), and cellular mechanisms (e.g., Schwann cells, stromal cells, T cells, and macrophages) [103].

Chemokines such as CCL2 [92], or endocrine hormones and neurotrophic factors, including nerve growth factor (NGF) and glial-derived neurotrophic factor (GDNF), are known to promote both in vitro and ex vivo culture models, suggesting that cancer cells surrounding nerves exhibit increased survival [100,101,104].

NGF, BDNF, neurotrophin 3, and neurotrophin 4, along with their receptors, are expressed in different PDAC cell lines, indicating a mutual influence between neurons and PDAC cells [103]. Notably, NTRK1 and NGFR are used as opposing prognostic markers for PDAC patients; NTRK1 expression is associated with a poor prognosis, whereas NGFR is linked to a longer survival rate [105].

PDAC cells rely on serine uptake, which is essential for tumor proliferation. Axons and dorsal root ganglia can secrete serine into the PDAC environment, providing energy support. After serine deprivation, PDAC cells release more NGF, enhancing the movement of axons toward the tumor [106]. Reducing the supply of serine or inhibiting NGF may be an attractive strategy for slowing the progress of neuroinvasion (NI) and tumor growth [107].

The GDNF family comprises GDNF itself, neurturin, artemin, and persephin, which can bind to multiple receptors and perform distinct functions [108]. PDAC patients with NI express higher levels of GDN, indicating the crucial role of GDNF in the neural invasion process [109]. GDNF induces polarization and invadopodia formation in cancer cells, enabling matrix degradation through the production of matrix metalloproteinases, thereby accelerating cell invasion [110]. GDNF targets the RET receptor and GDNF family receptor alpha 1 (GFRA1). The GDNF-RET-GFRA1 axis relies on the activation of KRAS, suggesting that classical oncogenes are essential for neuronal invasion [103].

As mentioned earlier, specific cytokines appear to play a role in neural invasion (NI). C-X3-C motif chemokine ligand 1, produced by neural cells, binds to C-X3-C motif chemokine receptor 1, located on the surface of PDAC cells, to activate integrins and G protein-coupled receptors [111]. Another example of the impact of cytokines on NI is the release of CXCL12 by dorsal root ganglia, which attracts PDAC cells by binding to CXCR4 [112].

Not only do complex pathways of the nervous and immune systems influence NI, but even the seemingly uninvolved factors in tumor progression, such as catecholamines, can also exacerbate it. In preclinical models, norepinephrine induces NI by activating the β2 adrenoreceptor, thereby stimulating nerve growth factor (NGF) secretion, which suggests a correlation between neurophysiological stress and the progression of PDAC [113,114].

Various cells involved in crosstalk in perineural invasion (PNI) infiltrate the pancreatic TME. Schwann cells are typical cells in peripheral nerves. Relations between Schwann cells and PDAC cells are inconsistent and ambiguous. Schwann cells interact with PDAC cells through membrane proteins, such as NCAM1 and myelin-associated glycoprotein. They could also promote the adhesion of PDAC cells to Schwann cells with Mucin 1. Lastly, Schwann cells induce structural alterations in PDAC cells [115,116,117]. Schwann cells communicate with PDAC cells through proteins such as L1 cell adhesion molecule (L1CAM), TGF-β, MMP2, and MMP9 [118,119]. Schwann cells communicate with PDAC cells and recruit macrophages from the bloodstream to the PNI site. Macrophages under the influence of CCL2 secreted by Schwann cells express cathepsin B, which degrades collagen IV, a significant component of perineurium, increasing PNI [120].

PSCs, mentioned above as the most prominent cell type in PDAC stroma, also contribute to PNI [121,122]. PSCs release tenascin C, enhancing the interaction between PDAC and dorsal root ganglia axons. Mutual relations between PDAC cells, neurons, and PSCs seem to be entangled with the SHH pathway [123,124].

TAMs also vaguely affect PDAC–neural cross-talk. Increased TAM invasion in PNI was associated with poor prognosis in PDAC patients [125]. Colony-stimulating factor 1 (CSF1), secreted by PDAC, recruits and activates macrophages, which in turn produce GDNF, thereby increasing PNI [126].

T cells—the main component of the adaptive immune system, which are essential for killing cancer cells—could be severely impaired by the parasympathetic nervous system. The acetylcholine (ACh) secreted by the vagus nerve was elevated in PNI-detected samples from PDAC patients. ACh suppresses CCL5 expression in PDAC cells, leading to insufficient T-cell infiltration. ACh also inhibits interferon-γ expression in CD8+ T cells, favoring Th-cell type 2 over Th-type 1 differentiation. Unfortunately, vagotomy’s impact on PDAC progression is still very concerning, as different studies show opposite results. Vagotomy can lead to prolonged survival in mouse models by increasing CD8+ T cells and elevating the Th1/Th2 ratio [127]. Simultaneously, it can also accelerate tumorigenesis, possibly due to the upregulation of TNF-α [128].

By combining all of these findings, some practical applications can be identified. Firstly, several studies have identified the proteins associated with PNI and their correlation with prognosis. These include membrane and intracellular proteins [129,130,131] (Table 2).

Neuronal invasion occurs in over 80% of PCs, and it can be observed at every stage of PDAC, including the early course. It is detected without lymphatic or hematic metastasis, and even in tumors smaller than 2 cm [98,132]. However, a full perineural invasion where cancer cells infiltrate the outer layer of nerves (epineurium) is not as common as in mouse models [98]. Recent studies have not shown perineural invasion, but have identified coexisting cancer cells surrounding the nerves in KRAS-based PDAC models [133,134].

One of the most important effects of NI and pancreatic hyperinnervation is severe pain linked to extrapancreatic nerve plexus invasion by the tumors growing toward the retroperitoneal space [135]. Severe pain is considered to be an adverse prognostic factor for survival [136,137]. PNI impacts OS prediction. Retrospective studies conducted over 20 years have shown that the postoperative survival of PDAC patients was negatively correlated with nerve infiltration and could be used as a prognostic factor [138,139,140,141,142,143].

NI-free patients have better long-term survival [144]. Not only the presence of NI is a predictive factor, but the depth of nerve infiltration is also. Patients with tumor cells infiltrating beyond the axon of nerves have shorter DFS and OS than those whose cancer cells do not reach the axon [145]. Therefore, NI is one of the conditions that require finding a personalized therapeutic protocol [146].

The most critical role in causing pain is linked to protease-activated receptor 2 (PAR-2)-mediated signaling [147]. A variety of proteases secreted by TME activates PAR-2. Despite the pain, PAR-2 regulates tumor proliferation, differentiation, and invasion [148]. Interestingly, recent studies uncovered an ominous mechanism responsible for suppressing pain response in PDAC and delaying diagnosis. Glial cells, specifically Schwann cells, can be activated in the earliest precursor stages of PC, and they are responsible for anti-inflammatory regulation, which helps alleviate pain [149,150]. IL-6 and CXCL12, secreted by cancer cells, are responsible for glia-mediated pain suppression [149]. Another study indicated a significant impact of the autonomic nervous system, with emphasis on adrenergic pathways, on NGF secretion [114].

As mentioned before, the rich expression of GDNF contributed to the NI in PDAC patients [98]. The level of GDNF and the accumulation of mast cells around intrapancreatic nerves are closely associated with neuropathic pain [135].

Severe pain could simultaneously worsen a patient’s life in numerous ways. It is an adverse prognostic factor for survival, as mentioned above. In addition, it increases the risk of drug abuse and influences the quality of life.

The influence of PNI occurrence on treatment choice is a valid question that many research groups seek to answer. Patients who received neoadjuvant chemotherapy had lower PNI rates, unlike those who did not [151]. Radiotherapy also decreases the PNI rate, as appropriate irradiation reduces GDNF release in the dorsal root ganglia [152]. Also, low-dose irradiation using iodine-125 inhibited PNI and tumor growth, decreasing pain occurrence [153]. The latest studies have shown that telomerase-specific oncolytic viruses can repress PNI, indicating a potential role in PDAC treatment [154].

## 5. Bacterial and Fungal Influence on PDAC Behavior and Treatment Response

Despite the significant impact of stromal cells on PDAC, it is reported that pancreatic neoplasms harbor a markedly higher number of fungi and bacteria compared to the healthy pancreas [155,156]. Microbiome ablation protects against pre-invasive and invasive PDAC [157]. Meanwhile, the transfer of bacteria from PDAC-hosting mice, but not controls, reverses tumor protection [157].

Microbiota influence the response to checkpoint inhibitor therapy in certain cancers, such as melanoma and lung cancer [158]. Bacterial ablation in PDAC-carrying mice, using antibiotics, reshapes the TME, inducing immune system activation and increasing sensitivity to immunotherapy [157]. It has been demonstrated that long-term survivors (LTS) exhibit higher microbiota diversity compared to short-term survivors (STS). Moreover, 25% of tumor-associated microbiota can be found in the gut, suggesting that changes in the intestinal microbiome can alter the tumor microbiome [155]. However, the specific mechanisms by which bacteria, such as Bifidobacterium, impact PDAC remain unknown. Fecal microbiota transplantation with stool from LTS in healthy control mice mirrors the recruitment of immune cells to the TME. It is not observed when stool is transplanted from STS recruitment to the TME [155]. Gut microbiome composition can enhance the effectiveness of cancer immunotherapy by modulating the immune system. For example, immunosuppressive populations treated with stool transplants from LTS patients were modulated by decreasing tumor infiltration by Tregs [159]. The elevated presence of three bacterial taxa, Saccharopolyspora, Pseudoxanthomonas, and Streptomyces, is noted in LTS patients [155]. Saccharopolyspora rectivirgula has been shown to play a role in inflammatory lung diseases, such as hypersensitivity pneumonitis [160]. The presence of Saccharopolyspora spp. could be responsible for generating a pro-inflammatory environment. However, its role in PDAC still needs to be explored [155].

One of the investigated mechanisms was the activation of aryl hydrocarbon receptor (AhR) by tryptophan metabolites produced by bacteria. TAMs, which express high levels of the AhR, develop inflammatory types of cancer with worse survival outcomes. AhR activation promotes the expression of IL-10 in TAMs and inhibits IFNγ expression in CD8+ T cells. Loss of the gut microbiome or exclusion of tryptophan from the diet mimics the deletion of AhR [161].

Tryptophan metabolite-indole-3-acetic acid (3-IAA) is a crucial amplifier of the response to chemotherapy in PDAC. Data from observing a small cohort of patients reveal a robust correlation between 3-IAA serum concentrations during chemotherapy and OS. Clinical trials aiming to raise 3-IAA serum concentration during chemotherapy ultimately increase survival in PDAC (NCT02077881) [162]. The easiest way to achieve this goal is to increase tryptophan dietary intake; however, it was effective only in the group of patients responding to chemotherapy, which is correlated with their microbiota constellation. Many taxonomically distinct bacterial species can produce 3-IAA, and further studies are needed to assess a narrow selection of the appropriate microbiota [163].

Recently, Gammaproteobacterium (GP), in addition to the rest of the TME-residing bacteria, has been found in human PDAC. GP can metabolize gemcitabine into its inactive form, suggesting a role for GP in gemcitabine resistance [164].

## 6. Repressing Immune Response via Microenvironmental Components

PDAC is known to be unresponsive to checkpoint inhibitors (ICIs) like atezolizumab or durvalumab, which bind to PD-L1, or pembrolizumab and nivolumab, which bind to PD-1, despite their high efficacy in other malignancies, such as non-small cell lung cancer (NSCLC), melanoma, or renal cell cancer (RCC) [165]. Although the molecular mechanism responsible for immunity to ICPis is unknown, it is probably related to the TME. This challenge drives researchers to identify the novel agents that can enhance the immune system’s ability to recognize and eliminate cancer cells [166].

A critical factor necessary to elicit efficacy in PDAC immunotherapy is high microsatellite instability (MSI-high) [167]. Unfortunately, only circa 1% of patients present an MSI-high phenotype [168], even though the FDA approved pembrolizumab to treat MSI-high and mismatch repair deficient patients [121]. Another experimental therapy proposed for tumor-bearing mice combines radiotherapy and PD-L1 immunotherapy. Radiotherapy upregulates PD-L1 in tumor cells, enhancing the response to PD-L1 blockade. A recent study revealed an innovative therapy strategy: combining radiotherapy with the administration of pembrolizumab and trametinib, an inhibitor of MEK1/2 [122,169].

A remarkably simple yet crucial obstacle that renders PDAC resistant to immunotherapy is the poor infiltration of immune cells into the PDAC TME [1,170]. Nevertheless, the indigent immunogenicity of PDAC may not be as indigent as previously observed. Recent studies have shown that the PDAC immune constellation may be much more sophisticated than previously thought. It seems that immune cells could comprise up to 50% of the PDAC cellular component, but only a few are anti-tumor effector cells [168,171].

CD3+ T-cells are a significant immune cell type in PDAC, predominantly occurring in the stroma of the cancer rather than within the cancer cell nest. They coexist with dendritic cells, Treg cells, B cells, and high endothelial venules within tertiary lymphoid structures. Their primary source is local proliferation instead of migration [172]. Additionally, CD4+ T cells are frequently present in the PDAC environment, whereas CD8+ T cells are less common, with accumulation varying between 7% and 30%. Moreover, these cells are functionally deficient, as they express various co-inhibitory molecules [172,173].

Among CD4+ T-cells, only the Th1 subset promotes anti-tumor response, as Th2 promotes tumor development. Notably, Th2 cells constitute a significant population of CD4+ T cells within PDAC tumors, and their concentration is higher than that of Th1 cells and FoxP3+ Treg cells [174]. These findings indicate one of the mechanisms responsible for immunotolerance and identify a potential target for future therapeutic strategies. For example, chemotherapy, especially gemcitabine, can deplete Treg cells, thereby enhancing cytotoxic and helper T cell responses and restoring the anti-tumor effects of Cd40 agonists and ICIs [175,176,177,178].

Bringing lymphocytes to the table, we cannot overlook Chimer Antigen Receptor T-cells (CAR-T) cells, one of the most extensively studied technologies in modern medicine. Various artificial gene design strategies targeting PDAC stroma are being explored, despite some difficulties. PDAC is a solid tumor, unlike hematological malignancies, where CAR-T therapy was primarily introduced, and it demands extra research to work correctly. The FDA’s approval of Amtagvi, dedicated to treating unresectable melanoma, brings hope for the future of tumor-infiltrating cell therapy [168,171]. Over fifty clinical trials involving CAR-T therapy in PDAC are currently being conducted, primarily targeting CEA, PSCA, and the endogenous MSLN epitope. Only one trial made it to phase 3, yet it was withdrawn (NCT04037241).

Another approach in PDAC treatment is the use of tumor vaccines. They may contain one or several tumor-associated antigens (TAAs) or tumor-specific antigens (TSAs). TAAs are overexpressed in proteins of cancer cells, which may also be present in some non-cancerous tissues, making them non-specific. TSAs, on the other hand, are expressed exclusively on tumor cells [179]. TSAs’ vaccines are highly specific, immunogenic, and avoid self-antigen-induced T cell tolerance [180]. One of the most promising approaches is the pancreatic cancer cell line-based vaccine, known as GVAX. GVAX contains irradiated allogeneic pancreatic cells that cannot grow and exhibit a wide range of PDAC antigens. So far, results have demonstrated that GVAX is safe, well-tolerated, and induces antigen-specific T-cell responses; nevertheless, it still does not show superiority to standard chemotherapy [179].

Intense TAM infiltration, especially secreting IL-10 M2 macrophages, is correlated with poor survival; thus, TAMs may be a promising therapeutic target for immunotherapy [136]. In PDAC, macrophages can be recruited from monocyte and tissue-resident sources [137]. The proliferation of TAMs could be driven by colony-stimulating factor-1 (CSF-1) produced by CAFs. CSF1 influences TAMs to express higher levels of p21, which is responsible for more inflammatory and immunosuppressive phenotypes [181]. Macrophages can mediate tumor immunosuppression by directly interacting with CD8+ T cells and, in a more indirect manner, by secreting immunomodulatory factors that alter the TME [182,183]. As such, preclinical and clinical studies have focused on TAM targeting, such as CD40 agonist treatment. Despite promising results in preclinical models, clinical success has yet to be demonstrated, suggesting more complex relationships in vivo [144,182,184].

CD47—known as the “don’t eat me” signal, which binds to a signal regulatory protein α (SIRPα)—is located on phagocytes and suppresses phagocytosis [144]. Preclinical studies have demonstrated that inhibiting CD47-SIRPα in vivo enhances the efficiency of PDAC clearance by macrophages, a phenomenon that correlates with the surface density of the CD47 receptor [145].

In PDAC, CD68+ tumor-infiltrating macrophages play a crucial role in expressing CD47, and their number correlates with the clinical features of PDAC patients and OS. CD163+ M2 macrophages are second to CD68+ macrophages investigated in estimating PDAC survival. They do not correlate with CD47 expression; however, high CD163+ infiltration is associated with tumor diameter and a high pT-stage. Anti-CD47 treatment resulted in changes in the TME, characterized by an increase in pro-inflammatory macrophages, a decrease in anti-inflammatory macrophages, and an increase in the number of intratumoral lymphoid cells [166].

The combined targeting of both CD47 and PD-L1 results in a synergistic inhibitory effect in MPC-83 cells, but not in Panc02 transgenic mice, likely due to the activation of different immune-activating genes [166].

The primary difference between mouse models and human PDAC is the T-lymphocyte to B-lymphocyte ratio, which is higher in human tumors. Despite finding that PDA-bearing mice had cancer cell-specific CD8+ cells, the mice, like human patients, do not respond to ICPIs. The control of PDAC growth via ICPis was achieved by depleting CAFs expressing the fibroblast activation protein (FAP). Three findings suggested that CXCL12 was responsible for the overexpression of FAP+ CAFs. Inhibition of CXCL12 resulted in T-cell accumulation and acted synergistically with PD-L1, thereby diminishing the growth of cancer cells [185].

To date, research on the PDAC immune milieu has primarily focused on TAMs, CAFs, and lymphocytes. Interestingly, currently, investigations increasingly indicate the role of neutrophils. Tumor-associated neutrophils (TANs) were found to have an unexpected positive correlation with CD8+ T-cells, which is surprising because it was previously thought that TANs hinder lymphocyte infiltration into the PDAC environment [173,186,187]. In Table 3, we summarize the TME components.

## 7. Clinical Trials

Regarding all various mechanisms influencing the relations between cancer cells and TME cells in PDAC, many clinical trials are taking place to find a partial solution to the poor outcomes of standard treatment regimens. We collect some of them in Table 4.

## 8. Conclusions

In conclusion, this paper summarizes the impact of the PDAC microenvironment on disease etiology, prognosis, and possible therapeutic options. The most crucial TME constellation components are immune cells, especially macrophages and lymphocytes, fibroblasts, bacterial, and fungal microflora, and neuronal cells. Depending on the particular phenotype of these cells, the composition of the microenvironment, and the cell ratio, patients can experience different disease outcomes and varying vulnerability to treatment approaches. Studies aiming at TME appear to be a significant future research path, as the microenvironment comprises approximately 90% [209,210] of the tumor mass. Unfortunately, many ongoing studies fail when it comes to applying in vitro or animal-based trials in human patients. Nevertheless, we hope that eventually we will cross the Rubicon, surpassing the infamous 11% five-year survival rate.

## Figures and Tables

**Table 1:** Examples of cells secreting cytokines and chemokines responsible for the activation of PSCs [16,21,22,23,24,25,26,27,28].

Type of Cells	Cytokines and Chemokines
Cancer cells	TGF-β FGF PDGF IGF-1 IL-1β IL-6
Acinar cells	TGF-β CTGF IL-1β TNF-α
PSC	TGF-β CTGF IL-10 PDGF IL-6 IGF-1 CXCL12
Inflammatory cells	IL-6 IL-10 TGF-β IL-6 IL-1β TNF-α IGF-1
Platelets	PDGF TGF-β

**Table 2:** Example of proteins associated with PNI.

		Expression	
		Upregulated	Downregulated
Correlation with PNI	Positive	BIRC5, CCND1, CDH1, CD74, COL6A3, CTHRC1, CXCR4, IL-13RA2, LRP1, L1CAM, MAP1LC3A/B, MDK, MYBL2, MYC, PODXL, PTN, SDC2, SDC3, SNCG, TIMP2, VGF, WASL	
	Negative	FAS, POP1	CADM4, HPGD

**Table 3:** Summary of TME components and their impact on tumor behavior, metastasis, and treatment response.

Component of TME	Description	Impact on Tumor Behavior	Impact on Metastasis	Impact on Treatment Response
PSCs	Quiescent cells in the exocrine pancreas; upon activation, they express markers like α-SMA and desmin and produce ECM components such as collagen and fibronectin.	Promote cancer cell proliferation and migration. Inhibit apoptosis.	Contribute to ECM remodeling, aiding tumor invasion and metastasis.	Contribute to chemotherapy and radiotherapy resistance.
CAFs	Heterogeneous cells responsible for ECM deposition, arise from fibroblasts, mesenchymal stem cells, or PSCs, and express markers like FAP, α-SMA, and vimentin.	Promote tumor growth, neovascularization, and invasion through the secretion of growth factors.	Enhance metastasis Via ECM remodeling and secretion of pro-invasive cytokines.	Suppress anti-tumor immune responses, affecting immunotherapy efficacy.
Tregs	CD4+CD25+FOXP3+ cells that suppress immune responses and maintain self-tolerance.	Impair anti-tumor immune responses, promoting tumor immune evasion.	Facilitate metastasis by suppressing immune responses to circulating tumor cells.	Correlate with poorer prognosis; suppress immune checkpoint responses.
MDSCs	Immature myeloid cells that are activated by cytokines like GM-CSF and IL-13.	Suppress anti-tumor immunity by activating Tregs and inhibiting CD8+ T cells.	Promote immune tolerance, aiding tumor cell survival in distant sites.	Limit the effectiveness of immunotherapies by suppressing T cell function.
TAMs	Monocytes that differentiate into M1 or M2 macrophages; M1 promotes inflammation, but M2 is more pro-tumor.	M1 promotes inflammation, M2 promotes tissue remodeling, immune suppression, and tumor progression.	M2 phenotype enhances metastasis by facilitating ECM remodeling and immune evasion.	M2 macrophages are linked to poor prognosis, suppressing response to treatment.
VM	Cancer cells form vessel-like structures independent of endothelial cells and angiogenesis.	Provides an alternative tumor blood supply, facilitating nutrient and oxygen supply to the tumor.	Complicates tumor vascular architecture and enhances metastasis by forming new blood pathways.	Linked to poor prognosis and chemotherapy resistance.

**Table 4:** Clinical trials encompassing some components of TME in PC.

Clinical Trial	Status	Tested Drug	Target	Additional Interventions	Short Description, Objective of the Study, Outcome, If Available
NCT06904378	Phase 1 (Finished)/ Phase 2 (Not yet recruiting)	Ontegimod	CD11b	Nab-Paclitaxel, Gemcitabine	An open-label phase I/II clinical trial of Ontegimod with gemcitabine and nab-paclitaxel in unresectable PDAC prior to future studies incorporating anti-PD1 checkpoint immunotherapy. Results are not available yet.
NCT06825546	Phase 2 (Recruiting)	Icaritin	TNF-α, IL-6, PD-L1	Nab-Paclitaxel, Gemcitabine	Regulating the tumor immune microenvironment by reducing the secretion of TNFa and IL-6 and inhibiting PD-L1 expression through decreasing MDSC proportion. Results are not available yet.
NCT06639724	Phase 1 (Recruiting)	Fostamatinib	Syk kinase	Nab-Paclitaxel, Gemcitabine	Fostamatinib is a Syk kinase inhibitor currently FDA-approved for chronic idiopathic thrombocytopenia purpura, but it has not been studied in PDAC. The investigators hypothesize that Syk inhibition reprograms macrophages to an immunostimulatory phenotype in the TME. Thus, Syk inhibition with fostamatinib in combination with chemotherapy could improve outcomes for patients with PDAC while having a favorable safety profile. Results are not available yet.
NCT06492915	Phase 2 (Recruiting)	Chiauranib	VEGFR2, VEGFR1, VEGFR3, PDGFRa and c-Kit, Aurora B kinase, CSF-1R	Nab-Paclitaxel, Gemcitabine	Chiauranib, which simultaneously targets VEGFR/Aurora B/CSF-1R, several key kinases involved in tumor angiogenesis, tumor cell mitosis, and chronic inflammatory microenvironment. Results are not available yet.
NCT06145074	Phase 2 (Recruiting)	Propanolol	β-adrenergic receptors		Assessment of density and subtypes of tumor-infiltrating lymphocytes, desmoplasia, adrenergic receptor expression, and spatial distribution of immune cells in the TME. Results are not available yet.
NCT05927142	Phase 1 (Recruiting), Phase 2 (Recruiting)	Rintatolimod	TLR3	Durvalumab	Immunotherapy effectiveness is improved by the agonist effect of Rintatolimod, which enhances dendritic cell maturation. Results are not available yet.
NCT05546853	Phase 1 (Active, not recruiting)	NP137	Netrin-1	FOLFIRINOX	Inhibiting Netrin-1 impedes EMT, thereby reducing tumor progression and metastasis. Results are not available yet.
NCT04493060	Phase 2 (Active, not recruiting)	Niraparib	PARP1, PARP2	Dostarlimab	This phase II trial studies how well niraparib and dostarlimab work in treating patients with germline or somatic BRCA1/2 and PALB2 mutated pancreatic cancer that has spread to other places in the body (metastatic). Results are not available yet.
NCT03727880	Phase 2 (Active, not recruiting)	Defacitinib	FAK kinase	Pembrolizumab	Evaluating if reprograming the TME by targeting FAK following chemotherapy can potentiate anti-PD-1 antibody. Results are not available yet.
NCT02651727	Phase 1 (Terminated)	Defacitinib,	FAK kinase (VS-4718)	Nab-Paclitaxel, Gemcitabine	The company’s decision to de-prioritize 4718 development.
NCT03085914	Phase 1 (Completed), Phase 2 (Completed)	Epacadostat,	Indoleamine 2,3-dioxygenase 1	Nab-Paclitaxel, Gemcitabine, FOLFIRINOX, Pembrolizumab, Pemetrexed, Cyclophosphamide, Carboplatin, Cisplatin,	Additional cohorts (i.e., the mandatory biopsy cohorts) were designed to evaluate changes in the TME in participants with any advanced or metastatic solid tumor who had progressed on previous therapy with a PD-1 or a PD-L1 inhibitor. Results are not available yet.
NCT02600949	Phase 1 (Recruiting)	Synthetic Tumoor-Associated Peptide Vaccine Therapy,	Personalized peptide vaccine targeting antigenes of PDAC tumor	Imiquimod, Pembrolizumab, Sotigalimab	A personalized peptide vaccine is developed from a patient’s tumor cells and blood to be used as a biological therapy. Biological therapies, such as personalized peptide vaccines, may attack tumor cells and stop them from growing or kill them. Results are not available yet.
NCT02565758	Phase 1 (Completed)	ABBV-085	LRRC15		Targeting leucine-rich repeat-containing protein 15 (LRRC15) using specific antibody-drug conjugates (ABBV-085) has the potential to improve the outcome of patients with LRRC15+ cancers of mesenchymal origin or stromal desmoplasia [188].
NCT03932565	Phase 1 (Status Unknown)	CAR-T therapy	FAP/Nectin-4		Targeting Nectin-4 transmembrane protein, which is highly expressed on the surface of breast cancer, bladder cancer, non-small lung cancer, and pancreatic cancer [189].
NCT03168139	Phase 1/Phase 2 (Completed)	NOX-A12,	CXCL12	Pembrolizumab	Olaptesed pegol (NOX-A12) targets a key chemokine in tumor TME—CXCL12, which is involved in the homeostasis of blood and immune cells. The hypothesis is that inactivation of CXCL12 by NOX-A12 makes pancreatic tumors more susceptible to immunotherapy [190].
NCT02765165	Phase 1/Phase 2 (Terminated)	USL311,	CXCR4	Lomustine	Terminated because of business reasons not related to safety.
NCT03277209	Phase 1 (Terminated)	Plerixafor,	CXCR4	Cemiplimab, Lomustine	Terminated due to slow accrual.
NCT02907099	Phase 2 (Completed)	BL-8040 (motixafortide)	CXCR4		Blocking CXCR4 with BL-8040 may stop the growth of tumor cells by blocking some of the enzymes needed for cell growth [191].
NCT00841191	Phase 1/Phase 2 (Completed)	Siltuximab	IL-6		IL-6 is one of the main agents responsible for inflammation. Blocking IL-6 with siltuximab was tolerated, but without clinical activity in solid tumors, including ovarian and KRAS-mutant cancers [192].
NCT00769483	Phase 1/Phase 2 (Completed)	MK-0646,	IGF1R	Gemcitabine, Erlotinib	MK-0646 and Ganitumab (AMG 479) are monoclonal antibodies, while Istiratumab is a bispecific antibody, with all three targeting IGF1R (with additional blockade of ErbB3 by Istiratumab), preventing its binding with IGF1 and IGF2 ligands and inhibiting downstream signaling pathways, such as Pl3K/Akt and MAPK, which are involved in promoting tumor cell proliferation and survival and resistance to apoptosis. All three trials did not show prolonged PFS or OS compared to standard chemotherapy regimens [193,194,195].
NCT02399137	Phase 2 (Completed)	MM-141 (istiratumab),		Nab-Paclitaxel, Gemcitabine	
NCT01231347	Phase 3 (Completed)	AMG 479 (ganitumab),		Gemcitabine	
NCT01383538	Phase 1 (Completed)	IPI-926	Hedgehog	FOLFIRINOX	IPI-926 is an oral SHH pathway inhibitor. The initial response rate was high, and patients receiving IPI-926 maintenance showed further declines in CA19-9 levels even after FOLFIRINOX discontinuation. However, the trial was closed early, as a separate phase II trial of IPI-926 + gemcitabine indicated the detrimental effects of this combination [196].
NCT01537107	Phase 1 (Completed)	Vismodegib		Sirolimus	Vismodegib is an SHH pathway inhibitor. SHH signaling is predominantly active in stromal cells rather than tumor cells, leading to desmoplasia and creating a dense TME. By inhibiting this pathway, vismodegib was supposed to remodel TME and enhance chemotherapy efficacy. However, all three studies showed that adding vismodegib did not enhance efficacy compared to standard chemotherapy regimens [197,198,199].
NCT01088815	Phase 2 (Completed)	Vismodegib		Nab-Paclitaxel, Gemcitabine	
NCT01195415	Phase 2 (Completed)	Vismodegib,		Gemcitabine hydrochloride	
NCT01485744	Phase 1 (Completed)	LDE225		FOLFIRINOX	LDE225, in combination with gemcitabine and nab-paclitaxel, was well-tolerated in patients with metastatic PDAC and has a promising efficacy after prior treatment with FOLFIRINOX. Quantitative MRI suggested that LDE225 causes increased tumor diffusion and works particularly well in patients with poor baseline tumor perfusion [200].
NCT02358161	Phase 1/Phase 2 (Completed)	LDE225		Nab-Paclitaxel, Gemcitabine	
NCT03472833	Phase 3 (Terminated)	High dose vitamin D	Vitamin D metabolism		Slow recruitment and patients lost to follow-up due to the COVID-19 pandemic.
NCT04524702	Phase 2 (Active, not recruiting)	Paricalcitol		Nab-Paclitaxel, Gemcitabine, Hydroxychloroquine	In preclinical models, the combination of paricalcitol and hydroxychloroquine has been shown to remodel TME by reducing stromal activation, decreasing cancer-associated fibroblasts, and enhancing immune cell infiltration. However, the efficacy of this regimen in the clinical trial was hard to establish due to the early termination of the trial due to COVID-19 and lower-than-expected enrolment. No published data on OS or PFS outcomes from this are available [201].
NCT04617067	Phase 2 (Completed)			Nab-Paclitaxel, Gemcitabine	Results are not available at the moment.
NCT03520790	Phase 1 (Terminated)				Phase II was not pursued due to futility based on the NAPOLI-3 therapeutic clinical trial results.
NCT03519308	Early Phase 1 (Terminated			Nab-Paclitaxel, Gemcitabine, Nivolumab	The accrual goal was unmet, and the drug manufacturer pulled support.
NCT03331562	Phase 2 (Completed)			Pembrolizumab	Paricalcitol did not improve pembrolizumab’s efficacy, likely related to its short half-life of only 5–7 h [202].
NCT03883919	Phase 1 (Completed)			5-Fluorouracil, Liposomal Irinotecan, Leucovorin	The study showed increased tumor vascularity, potentially enhancing treatment efficacy. However, more insight is needed [203].
NCT03307148	Phase 1 (Completed)	ATRA	Vitamin A metabolism	Nab-Paclitaxel, Gemcitabine	The study showed that the combination of ATRA and Nab-Paclitaxel + Gemcitabine was safe and tolerable, establishing the recommended phase 2 dose. Pharmacodynamic studies indicated stromal modulation consistent with the proposed mechanism of action [204].
NCT04241276	Phase 2 (Active, not recruiting)				No clinical outcomes such as OS or PFS are reported at this stage [205].
NCT04390763	Phase 2 (Terminated)	NIS793	TGF-β	Nab-Paclitaxel, Gemcitabine, FOLFIRINOX, Spartalizumab	The study was terminated early following the NIS793 treatment halt and urgent safety measure issued in July 2023, as the continued evaluation of Standard of Care alone will not support the original purpose of this phase 2 clinical trial.
NCT04935359	Phase 3 (Completed)			Nab-Paclitaxel, Gemcitabine	Results are not available at the moment.
NCT05417386	Phase 1 (Terminated)			FOLFIRINOX	NIS793 is no longer being developed.
NCT02734160	Phase 1 (Completed)	Galunisertib		Durvalumab, Gemcitabine	Median OS was 5.72 months, and PFS was 1.87 months. The study concluded that, while the combination was tolerable, its clinical efficacy was modest, indicating that future research might focus on earlier lines of treatment or patient selection based on predictive biomarkers for TGF-β inhibition [206].
NCT04327986	Phase 1/Phase 2 (Terminated)	M7824		Gemcitabine	The study closed to accrual due to the worsening risk-benefit ratio for participants receiving bintrafusp alfa (M7824).
NCT03451773	Phase 1/Phase 2 (Terminated)				The study was closed after one treatment-related death.
NCT03086369	Phase 1/Phase 2 (Completed)	Olaratumab	PDGF-α	Nab-Paclitaxel, Gemcitabine	The primary endpoint, OS, was not met: median OS was 9.1 months in the olaratumab arm versus 10.8 months in the placebo arm. Similarly, median PFS was 5.5 months with olaratumab compared to 6.4 months with placebo [207].
NCT02550327	Early Phase 1 (Completed)	Anakinra	IL-1	Nab-Paclitaxel, Gemcitabine, Cisplatin	Results are not available at the moment.
NCT04999969	Phase 2 (Active, not recruiting)	AZD0171	LIF	Nab-Paclitaxel, Gemcitabine, Durvalumab	Results are not available at the moment.
NCT02119663	Phase 3 (Terminated)	Ruxolitinib	JAK	Capecitabine	The safety committee found no safety issues, but recommended halting the study based on a lack of efficacy in a similar trial. The sponsor terminated the trial.
NCT02117479	Phase 3 (Terminated)				The study was terminated early based on the results of the planned interim analysis.
NCT03563248	Phase 2 (Active, not recruiting)	Losartan	Collagen and HA degradation	FOLFIRINOX	Results are not available at the moment.
NCT01821729	Phase 2 (Unknown status)				In this single-arm phase 2 trial of 49 patients, the R0 resection rate was 61% among all eligible participants, with a PFS of 17.5 months and OS of 31.4 months, which suggests that this total neoadjuvant approach may offer improved respectability and survival benefits in this patient population [208].
NCT02715804	Phase 3 (Terminated)	PEGPH20, nab-paclitaxel, gemcitabine	Hyaluronan acid		Sponsor decision.

## Abbreviations

The following abbreviations are used in this manuscript:

Treg	Regulatory T lymphocytes
CD4	Cluster of Differentiation 4
CD25	Cluster of Differentiation 25
FOXP3	Forkhead box P3
TGFβ	Transforming Growth Factor Beta
IL-10	Interleukin 10
IL-1	Interleukin 1
MDSC	Myeloid-Derived Suppressor Cells
M-MDSC	Monocyte-like Myeloid-Derived Suppressor Cells
PMN-MDSC	Polymorphonuclear Myeloid-Derived Suppressor Cells
CD8+	Cytotoxic T lymphocyte
NK	Natural Killer cells
PDAC	Pancreatic Ductal Adenocarcinoma
GM-CSF	Granulocyte-Macrophage Colony-Stimulating Factor
IL-13	Interleukin 13
HIFs	Hypoxia-Inducible Factors
TAMs	Tumor-Associated Macrophages
TNFα	Tumor Necrosis Factor Alpha
HIF2	Hypoxia-Inducible Factor 2
CAFs	Cancer-Associated Fibroblasts
myCAF	Myofibroblastic Cancer-Associated Fibroblasts
iCAF	inflammatory Cancer-Associated Fibroblasts
apCAF	Antigen-presenting Cancer-Associated Fibroblasts
ECM	Extracellular Matrix
CK-19	Cytokeratin-19
SOX9	SRY-Box Transcription Factor 9
FOLFIRINOX	Fluorouracil, Leucovorin, Irinotecan, Oxaliplatin
GnP	Gemcitabine and Nab-Paclitaxel
R-PDAC	Resectable Pancreatic Ductal Adenocarcinoma
BR-PDAC	Borderline Resectable Pancreatic Ductal Adenocarcinoma
MSI-high	Microsatellite Instability-high
PD-L1	Programmed Death-Ligand 1
MEK1/2	Mitogen-Activated Protein Kinase ½
CAR-T	Chimeric Antigen Receptor T-cells
CEA	Carcinoembryonic Antigen
PSCA	Prostate Stem Cell Antigen
MSLN	Mesothelin
ICIs	Immune Checkpoint Inhibitors
CXCL12	C-X-C Motif Chemokine Ligand 12
CCL2	C-C motif chemokine 2
NGF	Nerve Growth Factor
GDNF	Glial Cell-Derived Neurotrophic Factor
HGF	Hepatocyte Growth Factor
STAT3	Signal Transducer and Activator of Transcription 3
β2	Beta-2 Adrenergic Receptor
TME	Tumor Microenvironment
OS	Overall Survival
DFS	Disease-Free Survival
PAR-2	Protease-Activated Receptor 2
ICPs	Immune Checkpoints
TANs	Tumor-Associated Neutrophils
FAP	Fibroblast Activation Protein
ERK	Extracellular Signal-Regulated Kinase
TILs	Tumor-Infiltrating Lymphocytes
PC	Pancreatic Cancer
CK-19	Cytokeratin-19
SEER	Surveillance, Epidemiology, and End Results Programme
PSC	Pancreatic Stellate Cells
PDGF	Platelet-derived growth factor
FGF	fibroblast growth factor
CTGF	Connective tissue growth factor
αSMA	α-smooth muscle actin
IGF1	Insulin-like growth factor 1
VEGF	Vascular endothelial growth factor
FSP1	Fibroblasts-specific protein 1
ETM	Endothelial-to-mesenchymal transition
SDF-1	Stromal-derived factor-1
ROS	Reactive Oxygen Species
NOXs	NADPH oxidases
ER	Endoplasmatic Reticulum
siRNAs	Small interfering RNAs
SMA+	Alpha-smooth muscle actin
SHH,	Sonic Hedgehog Homolog
Ptch	Patched transmembrane protein
SMO	Smoothened transmembrane protein
GM-CSF	Granulocyte-macrophage colony-stimulating factor
TIMP	Matrix-metalloproteinases inhibitors
PDX	Patient-dervied xenografts
RA	Retinoid acid
ATRA	All-trans-retinoid-acid
HMGA1	High mobility group A1
ADM	Acinar-to-ductal metaplasia
GFRA1	GDNF family receptor alpha 1
PNI	Perineural invasion
L1CAM	L1 cell adhesion molecule
CSF1	Colony stimulating factor 1
ACh	Acetylcholine
AChR	Acetylcholine receptor
PAR-2	Protease-activated receptor 2
LTS	Long-time survivors
STS	Short-time survivors
3-IAA	Indole-3-acetic acid
GP	Gammaproteobacterium
NSCLC	Non-small cell lung cancer
RCC	Renal cell cancer
TSAs	Tumor-specific antigens
GVAX	Pancreatic cancer cell line-based vaccine
BIRC5	Baculoviral IAP Repeat Containing 5
CCND1	Cyclin D1
CDH1	Cadherin 1
CD74	Cluster of Differentiation 74
COL6A3	Collagen type VI alpha three chain
CTHRC1	Collagen triple helix repeat containing 1
IL-13RA2	Interleukin 13 receptor subunit alpha 2
LRP1	Low-density lipoprotein receptor-related protein 1
MAP1LC3A/B	Microtubule-associated protein one light chain three alpha/beta
MDK	Midkine
MYBL2	MYB proto-oncogene like 2
MYC	MYC proto-oncogene
PODXL	Podocalyxin-like protein
PTN	Pleiotrophin
SDC2	Syndecan 2
SDC3	Syndecan 3
SNCG	Synuclein gamma
TIMP2	Tissue inhibitor of metalloproteinases 2
VGF	VGF nerve growth factor
WASL	WASP-like actin nucleation-promoting factor
FAS	FAS cell surface death receptor
POP1	Processing of precursor 1, ribonuclease P/MRP subunit
CADM4	Cell adhesion molecule 4
HPGD	15-hydroxyprostaglandin dehydrogenase
CD11b	Cluster of differentiation 11b
VEGFR1	Vascular endothelial growth factor receptor 1
VEGFR2	Vascular endothelial growth factor receptor 2
VEGFR3	Vascular endothelial growth factor receptor 3
CSF-1R	Colony-stimulating-factor receptor 1
TLR3	Toll-like receptor 3
PARP1	Poly(ADP-ribose) polymerase 1
PARP2	Poly(ADP-ribose) polymerase 2
LRRC15	Leucine-rich repeat containing 15 protein
IGF1R	Insulin-like growth factor 1 receptor
HA	Hyaluronic acid
LIF	Leukemia inhibitory factor
FOLFIRINOX	5-Fluorouracil, Irinotecan, Oxaliplatin, Leucovorin
